# Observation of dose dependent intravaginal Prostaglandin E2 application in free farrowing sows during parturition – a pilot study

**DOI:** 10.1186/s40813-021-00208-z

**Published:** 2021-04-02

**Authors:** Alexander Grahofer, Ramona Bill, Heiko Nathues

**Affiliations:** Clinic for Swine, Department of Clinical Veterinary Medicine, Vetsuisse Faculty, University of Bern, Bremgartenstrasse 109a, CH-3012 Bern, Switzerland

**Keywords:** Farrowing duration, Piglet interval, Placenta, Piglet distress, Umbilical cord lesion

## Abstract

The duration of birth is an important factor influencing the survival of piglets and the health of sows. A prolonged parturition is usually treated with oxytocin, even though several undesirable side effects are described. Therefore, the aim of this study was to evaluate the safety and efficacy of Prostaglandin E2 (PGE2) of different concentrations as an intravaginal applied gel after the birth of the fourth piglet in sows. Twelve sows were randomly allocated to one of four treatment groups: Group I (control group) application of placebo gel; Group II application of 0.5 mg; Group III application of 1.0 mg PGE2; Group IV PGE2 application of 2.0 mg PGE2. Total duration of parturition (time between first piglet and last placenta), piglet interval before and after treatment and placenta expulsion duration (time between first and last placenta) were recorded, and each piglet was scored for meconium staining and vitality. Furthermore, stillborn piglets were categorized into ante-partum and intra-partum deaths.

A significant dose-dependent effect of PGE2 after the fourth piglet in a linear regression model with group I, II and III on the total duration of parturition and the placenta expulsion duration was detected. An increase of the PGE2 dosage from 0 to 1 mg significantly reduced the total duration of parturition (group I: 553.7 ± 114.2; group II:456 ± 167.9; group III: 284.7 ± 40.5; *p*-value: 0.02) and placenta expulsion duration (group I: 364 ± 120; group II: 289 ± 144.1; group III: 119 ± 46.13; p-value: 0.03). Although no further significant differences between the groups using ANCOVA and a linear regression model including all groups were detected, severe meconium staining in more than 10% of piglets was observed in group II and IV. Moreover, piglets of group IV showed oedematous and haemorrhagic umbilical cords, lethargy and anoxia after treatment, and intra-partum deaths were recorded.

The best outcome for the sows and piglets was achieved using the 1 mg PGE2 dosage, whereas the other dosages showed more negative side effects, impairing the health and welfare of the animals. The results of this study can be used for further studies with larger sample sizes.

## Background

The duration of parturition is an important factor for the survival of piglets and the health of the sow [1]. Due to a steady increase in litter size in modern pig production, the duration of parturition has increased leading to a negative impact on subsequent fertility in sows [2]. A prolonged duration of parturition increases the risk of postpartal disorders such as post-partum dysgalactia syndrome (PPDS) and retained placentae [3]. Furthermore, an increased duration of parturition results in an elevated number of still- or weak born piglets [2]. Therefore, a prolonged parturition is usually treated with oxytocin, a potent uterotonic agent, even though several undesirable side effects such as a higher prevalence of umbilical cord ruptures, meconium staining and weak or stillborn piglets have been described [4–6]. Due to these reasons, Prostaglandin E2 (PGE2) is used as alternative drug for the induction and assistance of labour in human medicine. PGE2 increases the uterine contractions in intensity but not in frequency unlike oxytocin, which increases intensity and frequency, thus leading to insufficient perfusion of placentae and fetuses [7–9]. Another very important benefit of PGE2 is the ripening of an unfavourable cervix [7–9]. In addition, PGE2 influences the endogenous oxytocin release and therefore has a direct contractile effect on uterine smooth muscle [9, 10]. All these actions result in an increase of Bishop’s score, which reflects the normal changes the cervix undergoes in parturition [11], more likely to enter labour, fewer failed birth inductions and less caesarean sections in humans [10]. As every drug provides side effects, seldom-occurring side effects of PGE2 are mainly gastrointestinal disorders such as diarrhoea [12, 13]. Hence, the effects of PGE2 would be an improvement for the swine industry. Therefore, the aim of this study was to evaluate the safety and efficacy of PGE2 as an intravaginally applicable alternative on the farrowing process of the sow. In order to elaborate the optimal dosage in daily practice, an adaptive dose response pilot study with three different concentrations (2 mg (human dosage), 1 mg and 0.5 mg) of PGE2 applied after the fourth piglet was conducted.

## Results

In total 12 sows of one farrowing batch, randomly assigned to one of four treatment groups, were included in the study. No significant difference between such parameters of the four treatment groups that had been used for stratification were observed at the beginning of the trial. The mean litter number of the sows was 4.4 (SD: ± 2.7), the mean body weight was 255 kg (SD: ± 42.7) and the mean back fat was 11.4 mm (SD: ± 3.7). The median observation interval during parturition was 7.9 min (Min: 1.5, Max: 10.2).

The statistical analysis using ANCOVA revealed no significant differences between the four groups in regards to the outcome variables. An overview of all parameters of the different groups with the *p*-values is provided in Table 1. However, a significant relationship between the Placebo, 0.5 and 1 mg PGE2 group in total duration of parturition and in the placenta expulsion duration was detected in the linear regression model. An increase of the PGE2 dosage from 0 to 1 mg significantly reduced the total duration of parturition (group I: 553.7 ± 114.2; group II:456 ± 167.9; group III: 284.7 ± 40.5; *p*-value: 0.02) and placenta expulsion duration (group I: 364 ± 120; group II: 289 ± 144.1; group III: 119 ± 46.13; p-value: 0.03). All other parameters did not differ significantly in the linear regression model with both data sets (Table 2).

**Table 1:** Descriptive statistics of the collected parameters in the peripartum period of the four different treatment groups containing three animals. No significant differences among the groups could be detected

	Placebo	0.5 mg PGE2	1.0 mg PGE2	2.0 mg PGE2	***p***-value
	Mean ± SD	Mean ± SD	Mean ± SD	Mean ± SD	
**Sow traits**					
Body weight (kg)	267.3 ± 27.7	250.3 ± 67.2	242.3 ± 55.3	260 ± 3 4.5	0.6456
Back fat (mm)	10.9 ± 3.3	13.1 ± 6.9	11.9 ± 1.3	9.7 ± 2.4	0.9933
Litter number (n)	4.6 ± 2.5	4.6 ± 3.5	4.3 ± 2.3	4.6 ± 2.8	0.7216
Litter weight (kg)	20.9 ± 1.6	21.1 ± 6.1	18.3 ± 1.6	28.5 ± 2.3	0.1141
Number of total born piglets (n)	16.7 ± 3.8	17 ± 4.4	14.3 ± 2.8	20.3 ± 0.6	–
Number of still born piglets Typ2 (%)	0	7.9 ± 2.8	0	8 ± 9.8	0.9015
**Farrowing traits**					
Farrowing duration (min)	296 ± 166.7	252.7 ± 137.6	139.7 ± 2.1	195.7 ± 109.9	0.2353
Total duration of parturition (min)	553.7 ± 114.2	456 ± 167.9	284.7 ± 40.5	404.7 ± 256	0.0883
**Piglets traits**					
Piglets interval (min)	18.1 ± 6.1	15.8 ± 6.0	10.8 ± 2.9	10.1 ± 5.2	0.3204
Piglets interval before treatment (min)	23.5 ± 8.8	11.5 ± 5.1	10.5 ± 4.1	10.7 ± 4.5	0.4685
Piglets interval after treatment (min)	16.2 ± 8.3	16.8 ± 6.4	10.9 ± 2.4	9.9 ± 5.3	0.1841
Number of meconium stained piglets Score 0 (%)	73 ± 8.2	31.7 ± 10.7	70.3 ± 19.1	52.3 ± 10.8	0.9701
Number of meconium stained piglets Score 1 (%)	29.7 ± 1 0.0	54.0 ± 19.3	23.3 ± 21.7	36.0 ± 5.3	0.8071
Number of meconium stained piglets Score 2 (%)	4.0 ± 3.6	12.3 ± 21.4	6.7 ± 6.5	11.7 ± 7.6	0.7403
**Placenta expulsion traits**					
Placenta expulsion duration (min)	364 ± 120	289 ± 144.1	119 ± 46.13	273.3 ± 191.8	0.5979
Time from last piglet to first placental part (min)	− 112.6 ± 119.1	−85.6 ± 114.0	26 ± 30.5	− 57.3 ± 37.5	0.7835
Time from last piglet to last placental part (min)	257.7 ± 230.7	203.3 ± 43.7	145 ± 42.0	209.0 ± 147.3	0.2185
	Median (Min-Max)	Median (Min-Max)	Median (Min-Max)	Median (Min-Max)	
**Sow traits**					
Number of still born piglets (n)	0 (0–1)	1 (1–3)	0 (0–2)	1 (0–4)	0.9526
Number of still born piglets Typ 1 (%)	0 (0–2)	0 (0–1.9)	0 (0–4.6)	0 (0–0)	0.9159
**Piglets traits**					
Reanimation of piglets (%)	0 (0–0)	0 (0–5)	0 (0–0)	5 (4.8–10)	0.9788
Umbilical cord lesions (%)	0 (0–0)	0 (0–10.5)	0 (0–18.8)	5 (0–23.8)	0.9132
**Placenta expulsion traits**					
Expelled placental parts (n)	5 (2–7)	6 (6–7)	5 (4–5)	8 (7–12)	0.7722

**Table 2:** Linear regression analysis for farrowing related traits using the Placebo, 0.5 and 1.0 mg PGE2 data set and the Placebo, 0.5, 1.0 and 2.0 mg PGE2 data set. Significant differences in the linear regression models are marked bold

	Models using PGE2 0, 0.5 and 1.0 mg	***P***-values	Models using PGE2 0, 0.5, 1.0 and 2.0 mg	***P***-values
**No. of sows**	9		12	
**Birth duration (min)**				
**Intercept (SE)**	307 (61)	< 0.01	267 (53)	< 0.01
**PGE2 conc.**	− 156 (95)	0.14	−53 (46)	0.27
**R**^**2**^	0.27		0.12	
**Parturition interval (min)**				
**Intercept (SE)**	565 (59)	< 0.01	491 (76)	< 0.01
**PGE2 conc.**	− 269 (92)	**0.02**	− 75 (67)	0.29
**R**^**2**^	0.55		0.11	
**Piglets interval after treatment (min)**				
**Intercept (SE)**	17.3 (3.2)	< 0.01	16.6 (2.5)	< 0.01
**2 conc.**	−5.3 (4.9)	0.3774	−3.6 (2.2)	0.13
**R**^**2**^	0.14		0.20	
**Piglets interval before treatment(min)**				
**Intercept (SE)**	21.7 (3.5)	< 0.01	18.7 (3.0)	< 0.01
**PGE2 conc.**	−12.9 (5.3)	0.05	−5.2 (2.6)	0.08
**R**^**2**^	0.46		0.30	
**Piglets interval (min)**				
**Intercept (SE)**	18.5 (2.6)	< 0.01	17.3 (2.2)	< 0.01
**PGE2 conc.**	−7.2 (3.9)	0.11	−4.1 (2.0)	0.06
**R**^**2**^	0.32		0.32	
**Placenta expulsion duration (min)**				
**Intercept (SE)**	379 (56)	< 0.01	303 (68)	< 0.01
**PGE2 conc.**	− 254 (87)	**0.03**	− 48.0 (59)	0.44
**R**^**2**^	0.53		0.06	
**Time from last piglet to first placental part (min)**				
**Intercept (SE)**	− 126.7 (48)	< 0.01	−85 (41)	< 0.01
**PGE2 conc.**	138.6 (75)	0.11	32 (36)	0.40
**R**^**2**^	0.33		0.07	
**Time from last piglet to last placental part (min)**				
**Intercept (SE)**	258 (67)	< 0.01	223 (59)	< 0.01
**PGE2 conc.**	− 113 (104)	0.32	−22 (51)	0.68
**R**^**2**^	0.14		0.02	

The quadratic expression of PGE2 concentration was not significant in any model

Although no other significant differences between the groups were detected, the mean farrowing duration and total duration of parturition was shorter in group III compared to all other groups. The mean total duration of parturition was 285 min in group III, and more than 400 min in all other groups.

**Fig. 1:**
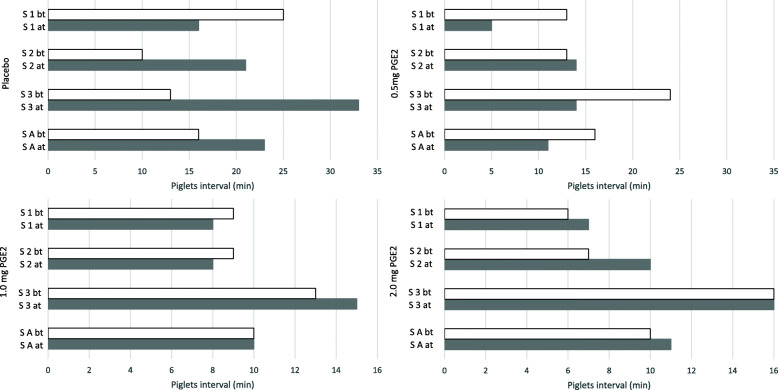
Interval between each piglet expulsion before (bt) and after treatment (at) in the four different treatment groups. The different light bars represent values for individual sows (S1 = sow 1; S2 = sow 2; S3 = Sow3) and the dark bars represent mean values (M) for each group

Despite the fact that differences were counted in the number of total born piglets per litter, the PGE2 groups had a shorter piglet-to-piglet interval, when compared to placebo. The shortest average piglet-to-piglet interval was observed in group IV, followed by group III with a prolongation of less than 1 min on average, and group II and group I with more than 4 min longer lasting intervals between two piglets. More detailed information about the piglet interval before and after treatment is provided in Table 1 and Fig. 1.

The shortest mean placenta expulsion duration was found in group III with more than 170 min difference compared to the other groups. Interestingly, group III and I had the same amount of placenta parts (median of five), whereas group II and IV had more than six placenta masses. Further information about the placenta expulsion traits is provided in Table 1.

No significant differences in the meconium score could be detected between the groups. The results of the different treatment groups regarding the meconium staining are presented in Table 1. In addition, umbilical cord lesions in live born piglets, such as oedematous and haemorrhagic umbilical cords, were observed in group II, group III and group IV only after the treatment, A reanimation procedure due to anoxia and lethargy was only needed in piglets of group II and group IV after the treatment.

## Discussion

This is the first study describing the effect of PGE2 in farrowing sows and the impact on piglets’ health during the farrowing process. The time point of application was chosen based on data of a study describing the influence of time at which oxytocin was administrated during labour in sows [14]. Administration of oxytocin after expulsion of the fourth piglet had the highest impact on the total duration of parturition and fewest side effects on the piglets’ health [14].

In order to evaluate the effect of PGE2, a pilot trail was conducted in sows balanced for parity, body weight and condition receiving a fixed dosage to efficiently gain more information about the dose response in farrowing sows. These data can be utilized for an informed decision, which dosage should be used for further investigations. The small sample size with only three animals per group is a major limitation of this pilot study. However, this study design has been chosen to obtain first and novel information about the dose response to PGE2 in sows [15–17].

Considering the limitations, this study showed a significant dose-dependent effect of PGE2 (Placebo, 0.5 and 1 mg PGE2) after the fourth piglet on the total duration of parturition and the placenta expulsion duration. No further significant differences were detected. Notwithstanding, the shortest farrowing duration and shortest total duration of parturition could be detected in the group III with a mean of 140 min and 285 min, respectively. Comparing the results with the group I (farrowing duration: 296 min; total duration of parturition: 554 min) and the farrowing duration of sows in free farrowing from literature, this is notably a very short duration of parturition, which has not been described so far. Taking the effect of PGE2 on duration of parturition into account, this might be a good alternative drug to oxytocin, because it is assumed to also decrease the risk for postpartal diseases in sows. Notwithstanding, the absolutely shortest piglet interval (9.9 min) has been observed in the group treated with the highest dose of PGE2, followed by group III (10.8 min), whereas the group with the lowest dose of PGE2 showed no effect compared to placebo. However, after administration of PGE2 the piglet interval was only reduced in six out of nine sows. The authors hypothesize that the reason for this result is linked to the free farrowing housing system, because sows have a higher average oxytocin concentration during the post-expulsion oxytocin pulses compared to crated sows [1]. Therefore, uterotonic drugs might have less effect in the normal birth process of loose farrowing sows.

Based on the findings of this study, we can hypothesize that PGE2 in the concentrations of 1 mg and 2 mg has an uterotonic effect in sows, as already observed in human medicine [10]. The intravaginal application route of PGE2, like in women, was chosen to improve animal welfare and to establish a safe and effective route of application that can be used by farmers, when accommodating free farrowing sows. The intravaginal administration was easy to handle and no anxiety or adverse reaction of sows during treatment were observed. Due to significant similarities between swine and human vaginal mucosa, the swine vaginal mucosa has been proven to be the gold standard as in vitro model for the transmucosal absorption of drugs [18]. Therefore, it can be assumed, that intravaginal applied PGE2-gel, which was designed for human medicine, can also be absorbed by swine mucosa. However, there is still a species difference and the absorption of certain agents might remain slightly unequal. An example has been described for oxytocin, where the transvaginal absorption is 53% more efficient in the sow compared to the absorption in human vaginal tissue [19]. In this pilot study a higher effect in sows when using the human dosage (2 mg PGE2) was observed, which might be related to the effect of absorption as mentioned.

The percentage of weak- and stillborn piglets are a major problem of intensive pig production systems and cause ethical discussions in the society [2]. Therefore, one outcome variable in this dose finding study was piglets’ distress. A proven indicator of intrauterine foetal distress is the meconium-stained skin of the piglets after birth [6]. In this pilot study, severe meconium staining was found in the placebo group and in all PGE2 groups. Some degree of distress caused by foetal hypoxia during parturition seems to be physiological due to compression of the umbilical cord when the foetus enters the pelvis. Severe meconium staining in piglets from sows with a placebo treatment were described in a study from Mexico in 1.1% of born piglets [6]. An explanation for this low percentage compared to group I might be that the average number of total born piglets was just 9.6 compared to 16.7 in the present study. Late born piglets are likely to suffer asphyxiation to a greater degree because of the cumulative effects of successive uterine contractions. These piglets have a greater predisposition to get in a hypoxia status which can provoke sever distress and thereby severe meconium-stained skin in piglets. Furthermore, piglets that take longer to be born are more likely to have umbilical cord lesions at birth [20]. The importance of an intact umbilical cord to improve piglets’ survival up to 3 days after parturition has been shown in another study [21]. In the present study, the highest percentage of umbilical cord lesion was recorded in group II. Interestingly, there was no association found between umbilical cord lesions and the meconium scoring. In comparison with other studies testing uterotonic substances [4, 5, 14, 22], we rarely found umbilical cord rupture, which can occur in up to 33% of normal deliveries. The main findings in the PGE2-groups were oedematous and haemorrhagic umbilical cords. Several factors can lead to such changes of the umbilical cord, occurring either prenatal or during the farrowing process and therefore is still under investigation in human medicine.

Further important clinical parameters for piglets’ vitality were ‘reanimation of piglets’ and ‘intra-partum deaths’. Both events were only detected in group II and IV. In group IV strong uterine contractions in the sows were observed by evaluating abdominal straining, which might have caused the high number of weak born piglets and intra-partum death. Strong uterine contraction was not observed in group II. The reason for this high number of intra-partum deaths in group II remains unclear and might be just by chance due to the small sample size. Severe hypoxia during delivery originates from the uterus, umbilical cord or placenta. In humans, placental dysfunction is considered the major cause of late foetal death [23]. Because the pig placenta is epitheliochorial, the need for an adequately intimate connection between sow and the piglets is met by the large total surface area of the diffuse placenta. However, there is few data available about the interaction between placenta expulsion and piglets’ vitality during parturition. An interaction may arise, when considering the relationship between the placental parts and the intrapartum death and reanimation in these groups, due to a placental dysfunction during parturition.

## Conclusion

For the first time, this pilot study evaluated different concentrations of Prostaglandin E2 (PGE2) with an intravaginal application route. Based on aspects of animal welfare, minimal required efficiency and safety of the product, the sample size was limited to three sows per group. This study revealed a significant dose-dependent effect of PGE2 towards 1 mg PGE2 after the fourth piglet on the total duration of parturition and the placenta expulsion duration with only minor sides effect on the piglet vitality. Taking these facts and the further results of this study into account, the best outcome for the sows and piglets is achievable with the intravaginal application of 1 mg PGE2 intrapartum after the birth of the fourth piglet. Further investigation is needed to confirm the positive effect of PGE2 in daily practise.

## Methods

Twelve crossbred (Large White x Landrace) sows from a satellite herd in Switzerland were included in this double blinded dose finding study. This study was conducted according to the Swiss law for Animal Welfare and approved by the cantonal veterinary office (Animal experiment license number: SO05/16). The sows were housed in a free farrowing system with pens being 2.15 × 2.60 m (i.e. 5.59 m2) and with partially slatted floors. The sows received a commercial diet (digestible energy 13.8 MJ/kg, crude protein 22.0%, crude ash 7.8%, crude fat 6.0%, crude fibers 5.2%) two times a day via an automated liquid feeding system and had unlimited access to water from a bowl drinker. All sows received straw as rooting and nest building material (approx. 1 kg/sow/day). The sows were stratified according to their litter number, backfat and body weight and randomly distributed into the four different treatment groups. To evaluate the effect of PGE2 on the parturition process, following treatment groups were established.

- Group I (control, *n* = 3) intravaginal application of 1.2 mL sterile gel (KY Jelly®, Johnson and Johnson)
- Group II (*n* = 3) intravaginal application of 0.5 mg (human dosage) of PGE2 in 1.2 mL gel (Prostin®E2 (Dinoprostone), Pfizer)
- Group III (*n* = 3) intravaginal application of 1.0 mg of PGE2 in 1.2 mL gel (Prostin®E2 (Dinoprostone), Pfizer)
- Group IV (*n* = 3) intravaginal application of 2.0 mg of PGE2 in 1.2 mL gel (Prostin®E2 (Dinoprostone), Pfizer)

The gel was stored in the refrigerator at 6 °C and before usage, it was warmed up by rolling the syringe in the hands. The body weight, litter number and the backfat thickness of sows were assessed, when they entered the farrowing unit approx. Seven days before the calculated date of parturition. Backfat thickness of the sows was measured at the P2-position (6.5 cm off the midline and over the last rib) using an ultra-sonographic device (iScan, Draminski, Poland). After the expulsion of the first piglet, the data collection for the parturition process started. At each observation, new-born piglets were recorded and each piglet was scored for meconium staining using a modified score (0 = piglet without meconium staining (Fig.1); 1 = piglet with slight meconium staining (< 2/3 of the piglet); 2 = piglet with severe (> 2/3 of the piglet) meconium staining) [5, 6] and vitality (condition of umbilical cord: intact; umbilical cord lesion such as oedematous and haemorrhagic umbilical cords; umbilical cord rupture; reanimation: Yes or No; reanimation was conducted if piglet was breathing irregularly and skin color was pale or blue). Furthermore, stillborn piglets were categorized into type one (greyish, oedematous piglet, ante partum death) or type two (fresh dead piglet, intra partum death) deaths [5]. Additionally, the expulsion of placenta parts that had been expulsed at once, were recorded. Within a maximum of 3 min after expulsion of the fourth piglet, the group specific treatment was applied. This time point was chosen, because administration of oxytocin after expulsion of the fourth piglet had the highest impact on the total duration of parturition and fewest side effects on the piglets’ health [14]. The gel was applied with a syringe in the cranial part of the vagina. The observation was conducted until expulsion of the last placenta, which was determined retrospectively. After parturition, total litter weight was recorded.

The following parameters were recorded or calculated with regard to each parturition: farrowing duration (time between first and last piglet), total duration of parturition (time between first piglet and last placenta), piglet interval (before and after treatment), placenta expulsion duration (time between first and last placenta), first placenta expulsion (expulsion of first placental part relative to last piglet) and last placenta expulsion (expulsion of last placental part relative to last piglet).

### Data recording and analysis

Data were collected using structured and standardised data collection forms. All data were entered into a spreadsheet program (Microsoft Office Excel 2016). Continuous variables, such as body weight, litter number, backfat thickness, litter number, litter weight, number of total born piglets, farrowing duration, duration of parturition, piglets intervals and placenta expulsion traits were first tested for normality and homogeneity of variance using the Shapiro-Wilk normality test. In all non-normal variates parameters a logarithmic transformation was conducted. If these assumptions were met, then differences among the four groups were evaluated using a one-way Analysis of Co-Variance (ANCOVA) model using total born piglets as a covariate. In addition, linear regression analysis for farrowing related traits were performed using the Placebo, 0.5 and 1.0 mg PGE2 data set and the Placebo, 0.5, 1.0 and 2.0 mg PGE2 data set as a continuous variable. This grouping was conducted, because sever sights effect of PGE2 at a dosage of 2 mg was noted. In the linear regression model, the total born piglets were included as an explanatory variable, because the litter size can affect the farrowing traits. The level of statistical significance was set to *p* < 0.05Data were analysed using NCSS 12 Data (NCSS 12 Statistical Software (2018). NCSS, LLC. Kaysville, Utah, USA, ncss.com/software/ncss).

## Data Availability

All the data are presented in the main paper and accompanying figures.
